# Melatonin Promotes the In Vitro Development of Microinjected Pronuclear Mouse Embryos via Its Anti-Oxidative and Anti-Apoptotic Effects

**DOI:** 10.3390/ijms18050988

**Published:** 2017-05-05

**Authors:** Xiuzhi Tian, Feng Wang, Lu Zhang, Pengyun Ji, Jing Wang, Dongying Lv, Guangdong Li, Menglong Chai, Zhengxing Lian, Guoshi Liu

**Affiliations:** National Engineering Laboratory for Animal Breeding, Key Laboratory of Animal Genetics and Breeding of the Ministry of Agriculture, Beijing Key Laboratory for Animal Genetic Improvement, College of Animal Science and Technology, China Agricultural University, Beijing 100193, China; tian7550@163.com (X.T.); vicent007@126.com (F.W.); df765@sina.com (L.Z.); jipengyun1989@gmail.com (P.J.); caylajingjing@gmail.com (J.W.); suxingdemogu@icloud.com (D.L.); 15600911225@163.com (G.L.); cml313@163.com (M.C.); lianzhx@cau.edu.cn (Z.L.)

**Keywords:** melatonin, mouse, pronuclear embryo, microinjection, anti-oxidation, anti-apoptosis

## Abstract

CRISPR/Cas9 (Clustered regularly interspaced short palindromic repeats) combined with pronuclear microinjection has become the most effective method for producing transgenic animals. However, the relatively low embryo developmental rate limits its application. In the current study, it was observed that 10^−7^ M melatonin is considered an optimum concentration and significantly promoted the in vitro development of murine microinjected pronuclear embryos, as indicated by the increased blastocyst rate, hatching blastocyst rate and blastocyst cell number. When these blastocysts were implanted into recipient mice, the pregnancy rate and birth rate were significantly higher than those of the microinjected control, respectively. Mechanistic studies revealed that melatonin treatment reduced reactive oxygen species (ROS) production and cellular apoptosis during in vitro embryo development and improved the quality of the blastocysts. The implantation of quality-improved blastocysts led to elevated pregnancy and birth rates. In conclusion, the results revealed that the anti-oxidative and anti-apoptotic activities of melatonin improved the quality of microinjected pronuclear embryos and subsequently increased both the efficiency of embryo implantation and the birth rate of the pups. Therefore, the melatonin supplementation may provide a novel alternative method for generating large numbers of transgenic mice and this method can probably be used in human-assisted reproduction and genome editing.

## 1. Introduction

In 1980, Gordon et al. first employed the pronuclear injection (PI) method to transfer exogenous genes into mouse embryos [1], and the technology has been widely used ever since. Currently, the CRISPR/Cas9 technology has become the most effective and convenient technology for “editing” genes in living cells, and it has displayed incomparable advantages over other gene editing techniques. Using CRISPR/Cas9 technology combined with microinjection has significantly improved the efficiency of gene modification in animals. For example, transgenic pigs and sheep have been produced [2,3]. The shortcoming of microinjection is that it can damage cytoplasm and thus affect the survival and development of embryos. The low efficiency of PI may have been a result of accumulated unfavorable conditions. For instance, embryos that were cultured in vitro, particularly microinjected embryos, faced a relatively high oxidative stress environment that did not occur in the in vivo condition [4].

Melatonin (*N*-acetyl-5-methoxytryptamine, MT) is produced in the pineal gland of vertebrates but also in many tissues and organs, including the ovaries, the testes, the uterus, the placenta, bone marrow, the retina, and the lens [5,6]. As a multifunctional molecule, melatonin modulates circadian rhythm, enhances immune functions, and regulates ovarian physiology and reproductive capacity in mammals [7,8]. In addition, melatonin is a potent free radical scavenger, and this activity is extended to its metabolites [9,10]. In addition to directly scavenging ROS, melatonin upregulates the gene expression of several antioxidant enzymes, such as superoxide dismutase (SOD) and glutathione (GSH), and inhibits pro-oxidative enzymes to reduce cellular oxidative damage [11], which appears to be essential for successful pregnancy [12].

Melatonin has crucial effects on embryo implantation and development [13]. The beneficial effects of melatonin on embryonic development are partially attributed to its ability to downregulate the expression of pro-apoptotic genes (Bax and caspase-3), upregulate the expression of an anti-apoptotic gene (Bcl-2), enhance intracellular glutathione, and reduce ROS production [14,15]. These changes lead to less apoptosis among blastocysts and improve the embryo quality and efficiency of embryo implantation [16]. Melatonin supplementation in a culture medium generated long-term beneficial effects on embryo development in murine [15,16,17], bovine [18,19,20,21,22], porcine [23,24,25,26,27], and ovine species [28]. However, no report has examined whether melatonin could improve the in vitro development of microinjected pronuclear embryos of mice. Therefore, the main purpose of this experiment is to study the influence of different concentrations of melatonin on the development of microinjected pronuclear embryos of mice under in vitro conditions and to explore the potential mechanisms.

## 2. Results

### 2.1. Effect of Melatonin on In Vitro Development of Microinjected Mouse Pronuclear Embryos

**Figure 1:**
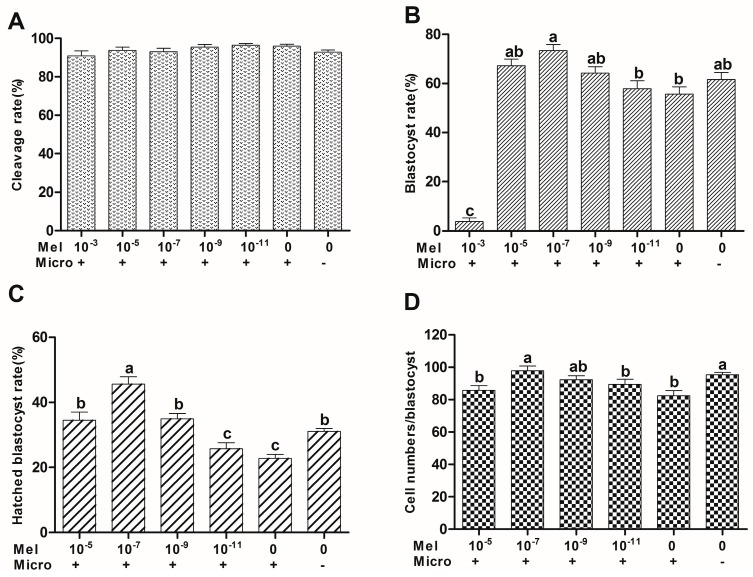
Effect of different concentrations of melatonin on in vitro development of microinjected mouse pronuclear embryos. Values 10^−3^, 10^−5^, 10^−7^, 10^−9^, 10^−11^, and 0 represent melatonin concentrations (M); + and − represents embryos microinjected and non-microinjected, respectively; Mel: melatonin; Micro: microinjected. (**A**) Cleavage rate; (**B**) blastocyst rate; (**C**) hatched blastocyst rate; (**D**) cell number/blastocyst. Means with different letters (a–c) in each column are statistically significant (*p* < 0.05).

The cleavage rates of microinjected pronuclear embryos treated with or without melatonin (control group) exhibited no significant differences with the non-microinjected control. The blastocyst rate of microinjected pronuclear embryos treated with melatonin at 10^−7^ M was significantly higher than the value of the control group (73.36 ± 2.54% versus 55.72 ± 2.90%) (*p* < 0.05). The blastocyst rates of microinjected pronuclear embryos treated with 10^−7^ M melatonin was even significantly higher than the non-microinjected control group (73.36 ± 2.54% versus 61.56 ± 2.92%) (*p* < 0.05). The hatched blastocyst rates at 10^−5^, 10^−7^, and 10^−9^ M melatonin groups are 34.51 ± 2.49%, 45.63 ± 2.26%, and 34.95 ± 1.64%, respectively, and these values were significantly higher than that of the control group (22.76 ± 1.22%) (*p* < 0.05). The hatched blastocyst rate of microinjected pronuclear embryos treated with 10^−7^ M melatonin was significantly higher than the value of the non-microinjected control group (45.63 ± 2.26% versus 31.08 ± 0.88%) (*p* < 0.05). However, melatonin at the highest concentration (10^−3^ M) significantly retarded embryo development and completely inhibited the formation of hatched blastocysts. The blastocyst cell numbers at 10^−7^ M melatonin group was significantly higher than that of the control group (97.85 ± 2.88 versus 82.35 ± 3.26) (*p* < 0.05). Detailed data were listed in Figure 1.

### 2.2. Effect of Melatonin on ROS in Embryos

**Figure 2:**
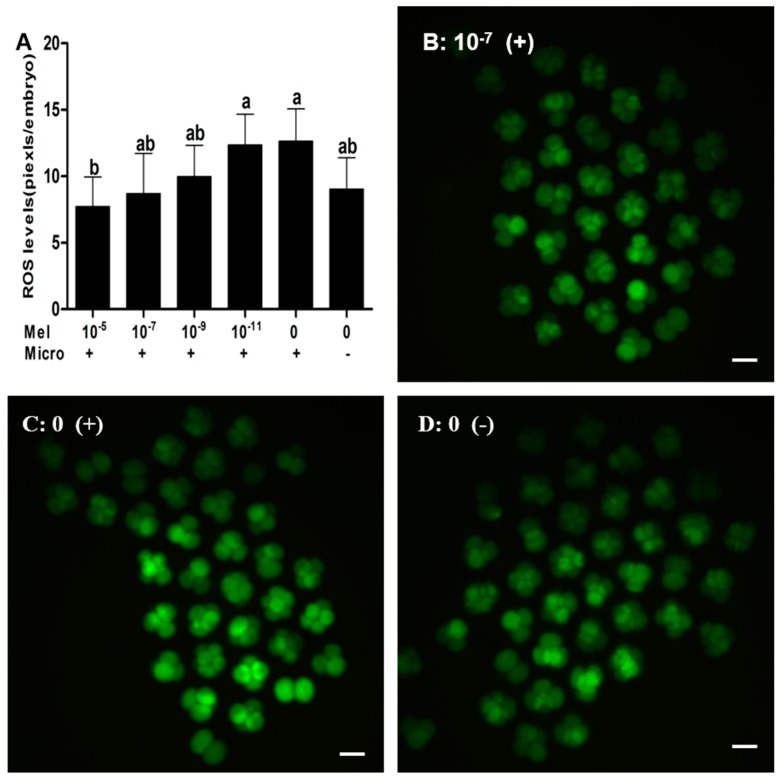
The effect of melatonin on ROS levels of microinjected mouse pronuclear embryos at 2-cell and 4-cell stages. Values 10^−5^, 10^−7^, 10^−9^, 10^−11^, and 0 represent melatonin concentrations (M); + and − represent embryo microinjection and non-microinjection, respectively. (**A**) Calculated reactive oxygen species (ROS) levels; (**B**–**D**) 2′,7′-dichlorohydrofluorescein diacetate (DCHFDA) fluorescence at 10^−7^ M melatonin, control, and non-microinjected embryo groups, respectively. Bars with different letters (a,b) indicate significant differences (*p* < 0.05). Abbreviations: Mel: melatonin; Micro: microinjection. Bar: 100 μm.

The levels of ROS in 2-cell or 4-cell embryos cultured in 10^−5^ M melatonin (7.73 ± 2.21 pixels/embryo) was significantly lower than that of the control group (12.64 ± 2.43 pixels/embryo) and the non-microinjected control group (9.04 ± 2.35 pixels/embryo). Moreover, the levels of ROS in the 10^−7^ M melatonin group (8.70 ± 3.02 pixels/embryo) were significantly lower than those of the control group (12.64 ± 2.43 pixels/embryo) but were not significantly different from the non-microinjected control group (9.04 ± 2.35 pixels/embryo). However, ROS in melatonin at 10^−9^ and 10^−11^ M groups exhibited no significant difference between the control and the non-microinjected control group. The data were illustrated in Figure 2.

### 2.3. The Effect of Melatonin on the Apoptosis of Blastocysts

**Figure 3:**
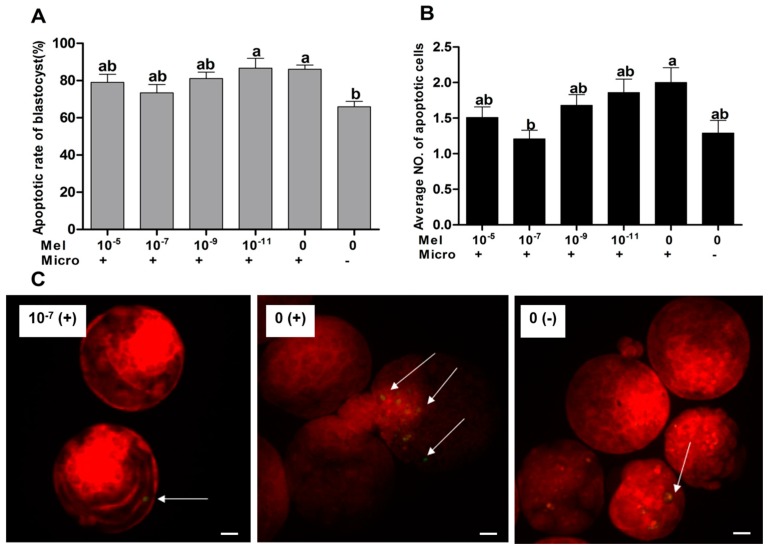
Effects of melatonin on apoptosis in mouse blastocysts via TUNEL. Values 10^−5^, 10^−7^, 10^−9^, 10^−11^, and 0 represent melatonin concentrations (M); + and – represent embryo microinjection and non-microinjection, respectively. White arrows: apoptotic cell. (**A**) Rate of apoptosis in blastocysts; (**B**) average number of apoptosis cells; (**C**) apoptotic cell staining, arrows pointed to the apoptotic cells). Bars with different letters (a–c) indicate significant differences (*p* < 0.05). Abbreviations: Mel: melatonin; Micro: microinjection. Bar: 20 μm.

The results showed that the apoptotic rates of blastocysts in 10^−5^ and 10^−7^ M melatonin-treated groups (79.55 ± 4.34% and 73.43 ± 4.42%, respectively) were significantly lower than that of the control group (86.15 ± 2.24%) (*p* < 0.05). The apoptotic rates in 10^−9^ and 10^−11^ M melatonin groups were significantly higher than the non-microinjected control group (81.14 ± 3.36% versus 65.53 ± 2.94%; 86.73 ± 5.23% versus 65.53 ± 2.94%) (*p* < 0.05). However, the 10^−3^ M melatonin group exhibited a significantly higher apoptotic rate (98.33 ± 1.20%) than did all of the experimental groups. The average apoptotic cell number/blastocyst in the 10^−7^ M melatonin group was significantly lower than that of the control group (1.21 ± 0.12 versus 2.00 ± 0.21) (*p* < 0.05) (Figure 3), and had no significance difference with the non-microinjected group.

### 2.4. The Effect of Melatonin on Pregnancy Rate, Birth Rate, and Litter Size after Blastocyst Implantation

**Figure 4:**
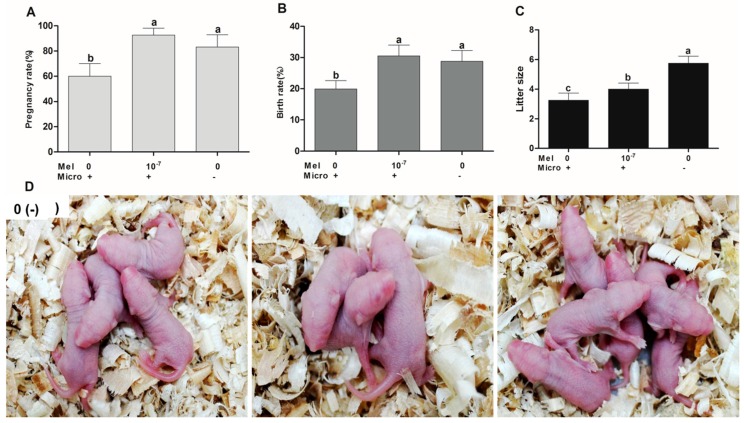
Effects of melatonin on mouse pregnancy and birth rates after blastocyst implantation. Values 10^−7^ and 0 represent melatonin concentration; + and − represent embryo microinjection and non-microinjection, respectively. (**A**) Pregnancy rate; (**B**) birth rate; (**C**) litter size; (**D**) the representative photos of the l litter size for different treatments. Bars with different letters (a–b) indicate significant differences (*p* < 0.05). Abbreviations: Mel: melatonin; Micro: microinjection.

The result showed that the pregnancy rate of the 10^−7^ M melatonin group was significantly higher than that of the control group (91.67 ± 5.37% versus 60.00 ± 10.00%) (*p* < 0.05). In addition, the birth rate of the 10^−7^ M melatonin group was significantly higher than that of the control group (30.51 ± 3.48% versus 19.94 ± 2.62%) (*p* < 0.05). The average litter size of the melatonin group was 4.00 ± 0.41 pups, whereas it was 3.25 ± 0.48 pups in the control group and 5.75 ± 0.48 pups in the non-microinjected control group (Figure 4).

## 3. Discussion

In the current study, we reported that melatonin supplementation at the concentration of 10^−7^ M significantly promoted the development of microinjected murine pronuclear embryos compared with the control group, as indicated by the elevated blastocyst rate, the hatched blastocyst rate, and the cell number/blastocyst. The blastocyst rate and hatched blastocyst rate at the 10^−7^ M melatonin group were even higher than those of the non-microinjected embryos. The cell number/blastocyst of microinjected pronuclear embryos with 10^−7^ M melatonin treatment was significantly higher than that of the control group, but was no different from that of the non-microinjected embryos. The beneficial effects of melatonin on the microinjected pronuclear embryos observed in this study were similar to those found in previous reports [17,23,29]. Most importantly, the pregnancy and birth rates in the melatonin-treated group were comparable to those in the non-microinjected embryos and significantly higher than those of the microinjected control group. An explanation for the relatively high pregnancy rate may be attributed to the improved quality of blastocysts treated with melatonin, which was indicated by the increased cell number of blastocysts. These blastocysts are likely more suitable for implantation and subsequently contributed to successful pregnancy. As a result, the high quality blastocyst also yielded more pups (Figure 4). The previous study showed that melatonin at 10^−9^ M significantly promoted blastocysts to implant and support pregnancy [16]. There was no report to show that melatonin could promote the birth rate of the cultured embryos, particularly the microinjected pronuclear embryos. This was observed in the current study (Figure 4).

During in vitro culture, embryos are exposed to relatively high oxidative stress compared to the in vivo environment, where the production of ROS in embryos is much lower [26], and high oxidative stress can cause severe damage to pre-implanted embryos. Embryos of mammals have relatively high levels of lipids, which make the mammalian embryos more susceptible to the deleterious effects of oxidative stress [30]. The elevated ROS levels in cultured embryos cause a loss of membrane integrity, alterations of structures and functions of proteins, and damage to nucleic acids [31]. These changes jeopardize the quality of in vitro cultivated microinjected pronuclear embryos and cause substantially low pregnancy and birth rates when these embryos are implanted into recipients. As mentioned previously, melatonin is a potent free radical scavenger and antioxidant, and the beneficial effects of melatonin on embryo development have been attributed to its effects on increasing intracellular glutathione, reducing ROS production, upregulating an anti-apoptotic gene (*Bcl-2*), and downregulating pro-apoptotic genes (*Bax* and *caspase-3*) [14,15,32]. Therefore, the supplementation of melatonin with a 10^−9^ M concentration lowered the apoptosis of blastocysts and improved both the embryo quality and the efficiency of embryo implantation [16]. It was reported that melatonin at 10^−7^ M increased blastocyst rates and blastocyst total cell number and reduced the apoptotic rate of parthenogenetic embryos in pigs, which is also attributed to its antioxidant capacity [33]. Similar effects were observed in the current study, and the suitable concentrations of melatonin for microinjected pronuclear embryo in vitro culture were identified as 10^−7^ and 10^−9^ M, which is consistent with parthenogenetic activation embryos [11,27]. Cellular apoptosis is a common pathological process that is associated with less than optimal culture conditions and oxidative stress during in vitro embryo development [26,34]. Melatonin treatment significantly retarded this pathological process, including reductions in apoptotic blastocysts and the average apoptotic cell numbers of blastocysts. In this study, we also observed the harmful effects of melatonin at a high concentration (10^−3^ M) on the development of the microinjected pronuclear embryo, which was consistent with the fact that 10^−5^ to 10^−3^ M melatonin was deleterious for embryo development, and confirmed the dual effect of antioxidant substances in in vitro reproductive techniques [35]. The main cause is attributed to melatonin’s pro-apoptotic and pro-oxidant actions, which is in agreement with previous research that showed that high concentrations of melatonin exerted pro-apoptotic effects on SH-SY5Y cells [36] and pro-oxidant actions on human leukaemia cells and inhibits cell division [37].

## 4. Materials and Methods

The study was carried out in strict accordance with the protocol approved by the Animal Welfare Committee of China Agricultural University (Permission Number: SYXK(Beijing)2015002; The period of valid days: 22 September 2015–22 September 2020). Pregnant mare serum gonadotropin (PMSG) and human chorionic gonadotropin (hCG) were purchased from Ningbo Hormone Products CO., LTD (Zhejiang, China). Melatonin and other reagents, unless specified, were purchased from Sigma Chemical Co. (St. Louis, MO, USA).

### 4.1. Embryo Collection

Kunming (KM) mice (China Experimental Animal Center of Military Medical Sciences, Beijing, China) of 8–12 weeks age were kept in a room with the temperature controlled at 20–22 °C under a 14:10 h light/dark cycle (lights on at 06:00 a.m.). After one week of acclimation, female mice were subjected to superovulation via an initial 10 IU PMSG injection (i.p.) and a 10 IU hCG injection (i.p.) 48 h later. Thereafter, the females were kept with sexually matured males overnight, and the females were then sacrificed by cervical dislocation. The pronuclear embryos were collected from the oviduct with M2 operating fluid, digested with 0.1% hyaluronidase, and washed 2 to 3 times with M2 fluid. Embryo handling was performed in the laboratory at ambient temperature (25 ± 0.5 °C). Media and embryos were maintained at 37 °C on a warming plate (Wenesco, Inc., Chicago, IL, USA) over the course of the operation. The pronuclear embryos were evaluated under a stereomicroscope, and normal embryos were selected for further study.

### 4.2. Pronuclear Microinjection and Culture

Microinjection was carried out describing in a standard protocol [38]. Pronuclear embryos with two clear pronuclear were chosen and used for microinjection. An Olympus IX-71 (Olympus, Tokyo, Japan) microscope equipped with the micromanipulator transgenic system was used for microinjection. Injection needles were prepared with a Sutter P-97 micropipette puller. Constant pressure microinjection was used for the pronuclear injection. Injection was terminated until pronuclear expansion, which was an indication of the 1–2 pL injection buffer (MR-095-F, Millipore Inc., Massachusetts, USA) being injected. After microinjection, pronuclear embryos with M16 medium (20–30 embryos/drop/50 μL) containing different concentrations of melatonin (0–10^−3^ M) in a Petri dish (35 mm × 10 mm, Corning Inc., Corning, NY, USA) were then cultured in an incubator (5% CO_2_, 37 °C, and 100% humidity). The in vitro survival of embryos was estimated by their development to the two-cell embryo stage at 24 h and then to the blastocyst and hatched blastocyst stages at 108–112 h, respectively. The blastocyst cell number was also calculated. To investigate the potential effect of melatonin on the in vitro development of microinjected murine pronuclear embryos, a total of 1480 pronuclear embryos were used.

### 4.3. Detection of ROS in Embryos

A total of 306 embryos were used to explore the effect of melatonin on intracellular ROS. Embryos were cultured in M16 medium supplemented with different concentrations of melatonin (0–10^−3^ M). After 24 h, the embryos at the 2–4 cell stage were collected to detect their intracellular ROS levels. Briefly, 2′,7′-dichlorohydrofluorescein diacetate (DCHFDA) (Beyotime Institute of Biotechnology, Jiangsu, China) was used as a green fluorescence indicator to detect intracellular ROS. A total of 25–30 embryos from each treatment group were incubated in DPBS-0.1% PVA containing 10 μM DCHFDA in the dark environment for 30 min, and the embryos were then washed three times with DPBS-0.1% PVA. The green fluorescence was measured using an epifluorescence microscope (TE300, Nikon, Tokyo, Japan) with UV filters (460 nm for ROS). The fluorescent images were saved as graphic files in TIF format. The fluorescence intensities of the embryos were analyzed using the ImageJ software (Version 1.40, National Institutes of Health, Bethesda, MD, USA) and normalized to that of the control embryos.

### 4.4. TUNEL Assay

A total of 276 murine blastocysts were stained by TUNEL. The blastocysts were washed 3–5 times with DPBS-0.1% PVA, fixed in 4.0% paraformaldehyde solution for 1 h, infiltrated in 0.1% Triton X-100 solution for 30 min, and exposed to a blocking solution at 4 °C overnight. Then, the blastocysts were incubated in TUNEL reaction solution (Roche, Indianapolis, IN, USA) at 37 °C for 1 h according to the manufacturer’s instructions and then stained with 50 μg/mL propidium iodide (PI) for 15 min. The apoptotic rate was calculated based on the ratio of the number of apoptotic blastocysts to total number of blastocysts and the average number of apoptotic cells per blastocyst.

### 4.5. Blastocyst Transfer

Based on previous embryo transfer methods [39], female mice that had been exposed to vasectomized males with the vaginal plugs were selected. The selected animals were anesthetized with pentobarbital sodium by intraperitoneal injection, and 12 blastocysts were then implanted into the uterine horns. A total of 216 blastocysts that were obtained from microinjected pronuclear embryos with 10^−7^ M melatonin incubation were transplanted into 18 recipient mice. In addition, a total of 180 blastocysts that were obtained from the microinjected control group were transplanted into 15 recipient mice and a total of 204 blastocysts that were obtained from the non-microinjected control group were transplanted into 17 recipient mice. Once embryo transplantation was completed, the mice were placed in a box with clean autoclaved sawdust. These recipient mice were handled with great care to avoid any stress because the pregnant mice are known to be easily stressed, which may lead to the abortion or even cannibalism of pups. The pups were born approximately 17 days after implantation. The pregnancy rates, birth rates, and litter size were recorded and statistically analyzed.

### 4.6. Statistical Analysis

The data are presented as the means ± SEM. The data were analyzed with a one-way analysis of variance (ANOVA) followed by Tukey’s method using SPSS 20.0 statistical software (SPSS Inc., Chicago, IL, USA). Statistical significance was set at *p* < 0.05.

## 5. Conclusions

In conclusion, melatonin in suitable concentrations (10^−9^ and 10^−7^ M) significantly improved the quality of microinjected pronuclear embryos in cultured conditions and increased the efficiency of embryo implantation and the birth rate of the pups. The mechanistic exploration identified the potential cause; that is, melatonin reduces oxidative stress and apoptosis in the cultured microinjection pronuclear embryos. These results suggest that the supplementation of melatonin can improve transgenic animal production efficiency in cultured microinjection pronuclear embryos. This may also be applied to somatic cell nuclear transfer and human-assisted reproduction, and these topics will be addressed in our future research.
